# Poisoning by Belladonna

**Published:** 1870-06

**Authors:** Chas. E. Hogeboom

**Affiliations:** Blackberry, Illinois


ARTICLE III.—*Poisoning by Belladonna.* By CHAS. E. HOGEBOOM, M. D., Blackberry, Illinois.

On the 14th of December, 1869, I was called in great haste, to see John Ramsey, of Blackberry, Illinois. The messenger said he was dying.

He was a patient of Dr. Pritchard, of Kaneville; suffering from phthisis, and confined to his bed. His condition when I first saw him at 6 1-2 P. M.: Insensibility complete, aphonia, loss of power of deglutition, irregular muscular contractions, pallor, pupil of eye greatly dilated, and insensible to light, pulse 138, respiration stertorous; in half an hour the face became red and swollen, eyes suffused with blood, pulse 160, very weak and intermittent, subsultus tendinum, coma.

He had taken nothing since 10 o'clock, A. M., except a fluid dram of Ext. Rhei, taken at 5 o'clock, P. M. Soon after taking this, he complained of dryness in the fauces, wanted some water; but could not swallow, and soon after became insensible.

Diagnosis: Belladonna poisoning.

℞ Morphiæ Sulph. gr.ss, every fifteen minutes; after several doses once in half an hour.

At first the morphia was put on the tongue to be absorbed; after three doses had been given, he could be made to swallow by the presence of fluid in pharynx.

We gave six or eight grains of morphia, with no other effect than a seeming relief of symptoms of poisoning. After each dose there would be an increase of irregular muscular contractions; but the pulse became slower and stronger. In about five hours he began to talk incoherently; in eight hours recognized some of his friends. He recovered.

Dr. Pritchard came about 10 o'clock, and shared responsibility in the case.

An analysis of the Ext. Rhei., fd., (so called) gave a large proportion of Belladonna. Ramsey must have taken about fifty drops of the fluid extract of Belladonna.

